# Serotonin Syndrome in the Emergency Department

**DOI:** 10.7759/cureus.6307

**Published:** 2019-12-06

**Authors:** Michelle Hernandez, Michael Walsh, Trilok Stead, Anines Quinones, Latha Ganti

**Affiliations:** 1 Emergency Medicine, University of Central Florida College of Medicine, Orlando, USA; 2 Pharmacy Department, Osceola Regional Medical Center, Kissimmee, USA; 3 Forensics, Trinity Preparatory School, Winter Park, USA; 4 Emergency Medicine, Envision Physician Services and Osceola Regional Medical Center, Kissimmee, USA; 5 Emergency Medicine, Envision Physician Services, Orlando, USA

**Keywords:** serotonin syndrome, clonus, neuroleptic malignant syndrome

## Abstract

With the widespread use of serotonergic agents including many antidepressants, antiemetics, illicit drugs, and even some herbal supplements, serotonin syndrome is a condition seen more frequently. It can appear abruptly and, if untreated, can progress to a life-threatening state. Prompt recognition and treatment is imperative to avoid complications. The presentation is variable and can be confused with other conditions. The authors present a case of serotonin syndrome that was recognized early and treated promptly in the emergency department.

## Introduction

Serotonin syndrome (SS) is an often undiagnosed and potentially life-threatening adverse drug reaction caused by excessive activation of postsynaptic serotonin receptors. The actual incidence of SS is difficult to measure because so many cases go unrecognized [1]. Reasons that contribute to SS being an elusive diagnosis include the following: the presentation of milder symptoms are presumed to be a general side effect of medication, unawareness of the syndrome as a clinical diagnosis, and the current variability of diagnostic criteria [1]. With the increasing widespread use of serotonergic agents in the general population, clinician awareness of this dangerous condition is essential.

## Case presentation

A 53-year-old male with a past medical history of hypertension, anxiety, and depression presented to the emergency department (ED) via emergency medical services with altered mental status. Earlier that day his wife found him confused and agitated with involuntary contractions of the lower extremities, so she called 911. He was given diphenhydramine and fluids prior to arrival. Upon arrival to the ED, he was oriented only to person, agitated, diaphoretic, hypertensive with a blood pressure of 170/84 mmHg and tachycardic at 109 beats per minute. Ocular clonus was noted along with inducible and spontaneous myoclonus of the lower extremities with hyperreflexia.

His wife provided a medication list which included bupropion, paroxetine, alprazolam, and zolpidem. Ten days prior, the patient was evaluated for back pain by his primary care physician and prescribed tramadol for his symptoms. He was instructed to stop taking his antidepressants and anxiolytics while taking tramadol. Two days prior to being evaluated in the ED he started taking all of his home medications, in addition to the tramadol for his back pain. His wife stated he was a math tutor and fully functional at baseline. She noticed some changes in his behavior after taking all the medications, but he acutely worsened the day of the ED visit. An extensive workup included a computed tomography (CT) of the brain without contrast, electrocardiogram, a complete blood count, and metabolic panel. The CT of the brain did not show any acute intracranial abnormality. Electrocardiogram revealed sinus tachycardia, normal PR and QRS intervals, but a prolonged QTc interval. Laboratory analyses revealed mild hypokalemia (Table 1).

**Figure 1:**
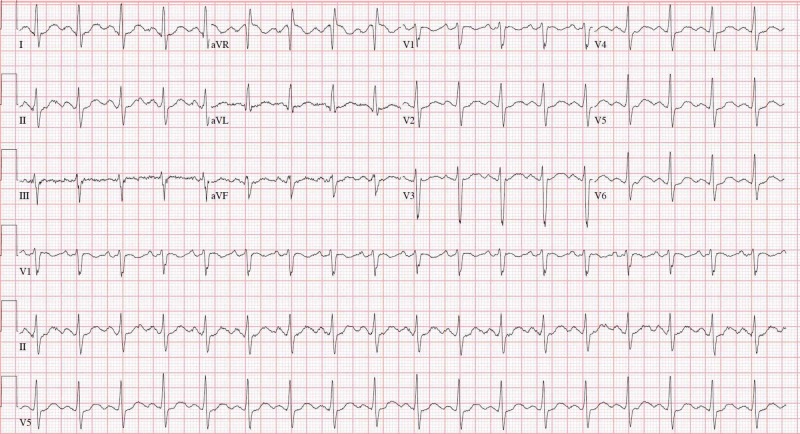
Electrocardiogram demonstrating sinus tachycardia and prolonged QTc interval.

**Table 1:** Laboratory results

Laboratory test	Value
White blood cell count	7.19 K/mm^3^
Hemoglobin	13.1 gm/dL
Hematocrit	40.70%
Platelets	292 K/mm^3^
Sodium	135 mmol/L
Potassium	3.1 mmol/L
Chloride	100 mmol/L
Calcium	9.6 mg/dL
Creatinine	0.79 mg/dL
Glucose	85 mg/dL

There was a high suspicion for SS given history of serotonergic medications with newly prescribed serotonergic drug, agitation, diaphoresis, ocular clonus, hyperreflexia, and clonus of lower extremities. Hunter’s criteria (see Discussion) were satisfied, and the critical care team was consulted for further management. He was given several doses of benzodiazepines in the ED and all serotonergic medications were withheld. Agitation improved with three doses of benzodiazepines. His symptoms improved, and he returned to baseline within 24 hours of treatment. All serotonergic medications were discontinued, and he was then discharged three days later.

## Discussion

SS is primarily manifested through its effects on the central nervous system and is characterized by a clinical triad of altered mental status, signs of neuromuscular irritability, and autonomic instability. It can occur secondary to an intentional or accidental overdose with a single serotonergic agent. However, most severe cases occur as a result of the interaction between two or more drugs that enhance serotonin transmission. The presentation is extremely variable, ranging from mild symptoms to life-threatening extremis. Symptoms tend to usually manifest within 24 hours of an increased dose of a serotonergic agent, addition of another serotonergic agent to a patient’s medication regimen, or after an overdose. Mild cases of SS will present with mild hypertension and tachycardia, mydriasis, diaphoresis, shivering, tremor, myoclonus, and hyperreflexia [1]. These patients tend to be afebrile and clinically stable. A moderate severity will have the above symptoms plus hyperthermia (40⁰C), hyperactive bowel sounds, horizontal ocular clonus, mild agitation, mania, and pressured speech. In severe cases, patients will have all of the above symptoms plus severe hyperthermia greater than 41.1⁰C, dramatic swings in pulse rate and blood pressure, delirium, and muscle rigidity. This clinical severity can result in seizures, rhabdomyolysis, metabolic acidosis, renal failure, respiratory failure, diffuse intravascular clotting, coma, and even death.

SS shares many common features with neuroleptic malignant syndrome (NMS), such as diaphoresis, elevated blood pressure, and altered mental status. It is important to distinguish between the two conditions as the treatment is different. However, they can be distinguished in many ways. NMS is triggered secondary to dopamine receptor blockade, frequently associated with antipsychotic and antiemetics use, while SS is due to excess serotonin receptor activation. NMS and SS can be distinguished in several ways [2]. Development of NMS tends to gradual, whereas SS tends to develop rather abruptly. NMS can also last from days to weeks, while SS tends to rapidly resolve. The core neuromuscular findings in each presentation also differ. The key physical exam finding in NMS is muscular rigidity, classically described as “lead-pipe rigidity,” whereas the hallmark physical exam finding in SS is spontaneous myoclonus, tremor, shivering, and increased tone in the lower extremities due to neuromuscular activation. Reflexes are normal in NMS, whereas they are increased in SS. Abdominal auscultation in NMS reveals hyperactive bowel sounds, whereas in SS they are normal or quiet. In NMS, there is noticeable mydriasis, while in SS, there is no noticeable pupil dilation.

SS is a clinical diagnosis of exclusion. There is no single diagnostic test available for confirmation, leading to the diagnostic gold standard being a diagnosis by a medical toxicologist [3]. However, if SS is suspected, diagnosis must occur rapidly due to the morbidity and mortality associated with this condition. Several sets of diagnostic criteria have been developed to help define SS. Three sets of criteria have been validated for diagnosis: Sternbach, Hunter, and Radomski [4]. The Hunter criteria are the most accurate diagnostic set available to diagnose SS with a sensitivity of 84% and specificity of 97% [5]. To fulfill the Hunter criteria, a patient must have taken a serotonergic agent and meet ONE of the following presenting symptoms:

• Spontaneous clonus

• Inducible clonus PLUS agitation or diaphoresis

• Ocular clonus PLUS agitation or diaphoresis

• Tremor PLUS hyperreflexia

• Hypertonia PLUS temperature above 38⁰C PLUS ocular clonus or inducible clonus

**Figure 2:**
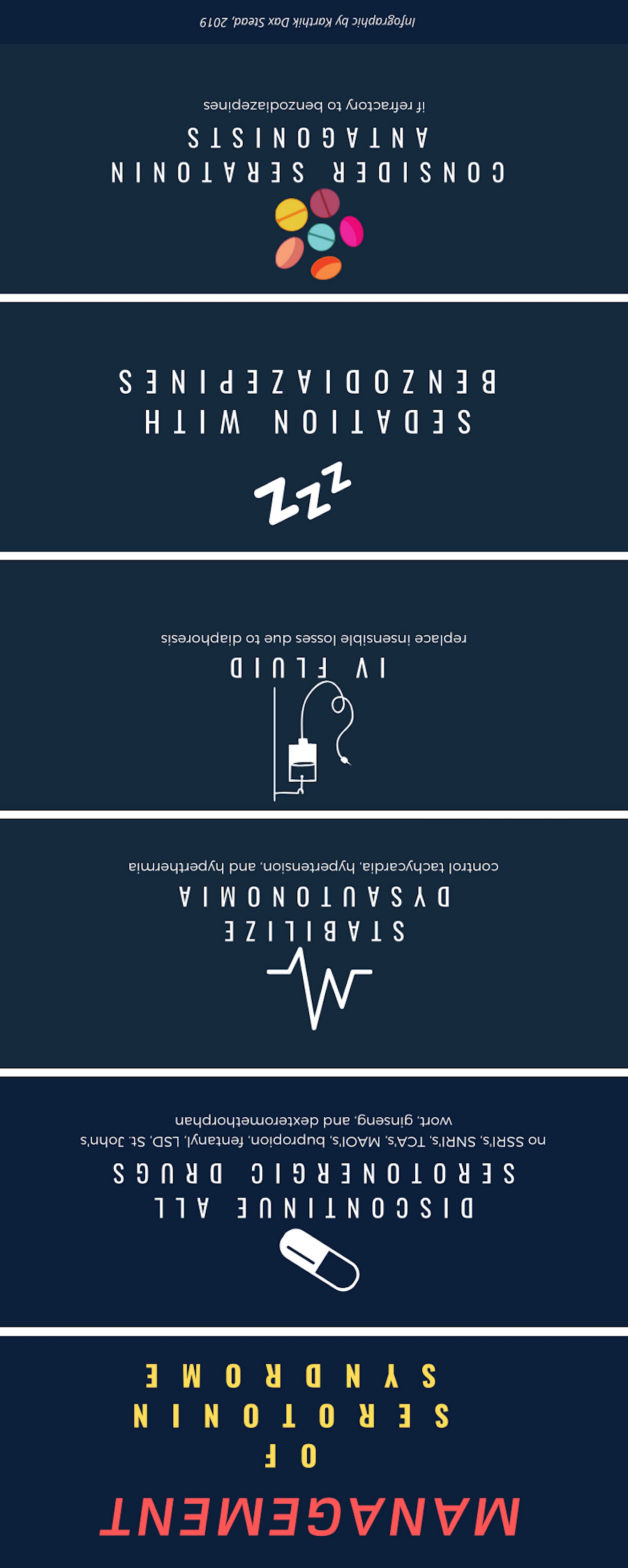
Management of serotonin syndrome SSRI, selective serotonin reuptake inhibitor; SNRI, serotonin–norepinephrine reuptake inhibitor; TCA, tricyclic antidepressant; MAOI, monoamine oxidase inhibitor; LSD, lysergic acid diethylamide

Mild-to-moderate cases of SS usually resolve in one to three days after stopping the serotonergic drugs, whereas a severe patient presentation is a medical emergency and requires rapid and intensive supportive care. The keys to management are discontinuing all serotonergic agents, stabilizing vital signs and oxygen saturation, administering intravenous fluids, providing continuous cardiac monitoring, initiating sedation with benzodiazepines, and possibly administering serotonin antagonists if supportive care and benzodiazepines fail (figure 2) [6].

Serotonin antagonists include cyproheptadine, olanzapine, and chlorpromazine. These agents have 5-HT2a antagonistic activities, although data are both lacking and conflicting in support for their use in SS management [7-9]. With these treatment principles, SS can resolve within as early as 24 hours.

## Conclusions

SS is a potentially life-threatening condition when not recognized promptly. The mainstay of treatment consists of withdrawing the offending (serotonergic) agent, supporting hemodynamic status, and administering benzodiazepines for sedation. Numerous serotonergic agents, including illicit substances, are now in widespread use; thus, maintaining a high level of suspicion for this syndrome is important. 
